# Flying MOFs: polyamine-containing fluidized MOF/SiO_2_ hybrid materials for CO_2_ capture from post-combustion flue gas†
†Electronic supplementary information (ESI) available. See DOI: 10.1039/c7sc05372j


**DOI:** 10.1039/c7sc05372j

**Published:** 2018-04-11

**Authors:** Ignacio Luz, Mustapha Soukri, Marty Lail

**Affiliations:** a RTI International , Post Office Box 12194, Research Triangle Park , NC 27709 , USA . Email: msoukri@rti.org

## Abstract

Solid-state synthesis ensures a high loading and well-dispersed growth of a large collection of metal–organic framework (MOF) nanostructures within a series of commercially available mesoporous silica. This approach provides a general, highly efficient, scalable, environmentally friendly, and inexpensive strategy for shaping MOFs into a fluidized form, thereby allowing their application in fluidized-bed reactors for diverse applications, such as CO_2_ capture from post-combustion flue gas. A collection of polyamine-impregnated MOF/SiO_2_ hybrid sorbents were evaluated for CO_2_ capture under simulated flue gas conditions in a packed-bed reactor. Hybrid sorbents containing a moderate loading of (Zn)ZIF-8 are the most promising sorbents in terms of CO_2_ adsorption capacity and long-term stability (up to 250 cycles in the presence of contaminants: SO_2_, NO_*x*_ and H_2_S) and were successfully prepared at the kilogram scale. These hybrid sorbents demonstrated excellent fluidizability and performance under the relevant process conditions in a visual fluidized-bed reactor. Moreover, a biochemically inspired strategy for covalently linking polyamines to MOF/SiO_2_ through strong phosphine bonds has been first introduced in this work as a powerful and highly versatile post-synthesis modification for MOF chemistry, thus providing a novel alternative towards more stable CO_2_ solid sorbents.

## Abstract 2

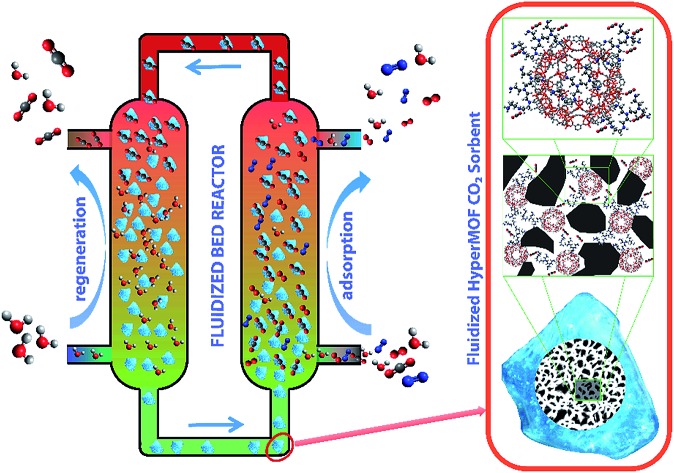
Solid-state synthesis ensures a high loading and well dispersed growth of a large collection of metal–organic framework (MOF) nanostructures within a series of commercially available mesoporous silica allowing to render MOFs into fluidized solid sorbents for CO_2_ capture from post-combustion flue gas in a fluidized-bed reactor.

## Introduction

CO_2_ capture from both coal- and natural gas-fired power plants using solid sorbents has been proposed as the most promising alternative to current amine-solution scrubbing technologies because of their lower energy requirements for sorbent regeneration, as well as less amine evaporation and equipment corrosion.^1^ The state-of-the-art for CO_2_ solid sorbents from post-combustion flue gas is mainly based on polyamines containing more than two ethylamine monomers (*i.e.* diethylenediamine, tetraethylenepentamine or polyethyleneimine) either (1) confined or polymerized *in situ* from aziridine within mesoporous silica cavities^2^ or (2) coordinated to the open metal sites of metal–organic frameworks (MOFs),^3^ which are preferred among supports, including zeolites, polymers or activated carbons.^4^

In these solid composites, polyamines selectively adsorb CO_2_*via* chemisorption at primary and secondary amines. MOFs also have the capability to absorb CO_2_*via* physisorption, either at Lewis-acid open metal sites^5^ or *via* molecular size confinement even in the presence of H_2_O,^6^ which have both demonstrated excellent capacity and selectivity over N. However, CO_2_ physisorption is reduced or limited by temperature, which leaves polyamine-impregnated MOFs as the most suitable strategy involving MOFs to prepare solid sorbents for CO_2_ capture under flue gas conditions typically operating at temperatures 40 °C and greater, although some promising results have been recently reported.^7^

In the race for developing more energy-efficient and competitive processes involving amine-containing solid sorbents for CO_2_ capture from post-combustion flue gas streams, fluidized-bed reactors (FBRs) are proposed as a more energy-efficient CO_2_ capture technology than packed-bed configurations due to their improved heat and mass transfer.^8^ In this way, several polyamine-containing solid sorbents using mesoporous silica as fluidized supports have been successfully tested in an FBR at pilot plant scale.^9^ Nevertheless, the current fluidized solid sorbents show capacities below 10 wt% of CO_2_ capture and suffer long-term deactivation *via* leaching of the active adsorbent phase because of the weak interactions *via* hydrogen bonds between polyamines and the support.

To the best of our knowledge, MOF-based CO_2_ solid sorbents have never been studied in a fluidized-bed configuration because of the absence or limitation of some essential features required for operating under these conditions, such as fluidizability and attrition resistance. Therefore, it is quite important to develop a general strategy for shaping MOFs into a fluidized form, which may confer MOFs with the required physicochemical properties. Furthermore, additional synergistic features can originate from MOFs and support intimately combined at the nanoscale, such as hierarchical mesoporosity or microporosity, which may host and disperse more active polyamines while providing them with more long-term stability *via* either a coordination bond at open metal sites or a covalent bond at free functional groups.^10^

Our group has recently developed an elegant approach to selectively confine MOF nanocrystals within mesoporous materials *via* novel solid-state synthesis, which allows the preparation of hybrid materials containing a high loading (up to 40 wt%) of the smallest nanocrystals yet reported for any MOF structure within a wide collection of mesoporous supports, also named HyperMOFs.^11^ The resulting hybrid materials show improved features from the combination of a silica scaffold protecting and supporting the MOF nanocrystallites within its porous network. Some of these improved features include improved attrition resistance, excellent fluidizability and handling, as well as the enhanced catalytic activity recently reported by our group.^12^ This insight encouraged us to prepare a novel kind of “flying” MOF by using cheap, commercially available mesoporous silica as the fluidizable carrier for MOFs.

Herein, a collection of polyamine-impregnated MOF/SiO_2_ was tested for CO_2_ capture under simulated flue gas conditions in a packed-bed reactor (PBR) and compared with the state-of-the-art sorbents composed of polyamines on both “fluidized” silica and “non-fluidized” bulk MOFs. This systematic study reveals the correlations between the features of MOF/SiO_2_, such as loading, composition, and functionality of the confined MOFs, and the performance of impregnated polyamines for CO_2_ capture. To study their viability for a larger scale, the most promising materials have been evaluated for long-term stability in a PBR and a visual fluidized-bed reactor (vFBR). In addition, a cost evaluation study was conducted by our group to compare with those of state-of-the-art MOF-based solid sorbents. Furthermore, a novel strategy for covalently bonding polyamines to the fluidized MOF/SiO_2_ hybrid materials was introduced during this work to precisely tailor the new generation of HyperMOF hybrid materials for specific applications.

## Experimental

### Fluidized MOF/SiO_2_ hybrid materials synthesis

The preparation and characterization steps of all the MOF/SiO_2_ hybrid materials included in this work were conducted by following our recently reported procedure for the solid-state synthesis of MOFs within mesoporous materials.^11^ Moderate concentrations of MOFs on SiO_2_ were also achieved by incipient wetness impregnation of the MOF precursor in dimethylformamide (DMF) or MeOH, as described in the ESI.†

### Bulk MOF synthesis

Bulk MOFs, such as (Cr)MIL-101(SO_3_H),^13^ ZIF-8,^14^ (Zr)UiO-66(NH_2_),^15^ Mg_2_(dobpdc),^16^ and NbOFFIVE-1-Ni,^17^ included in this work were prepared by following the reported procedures.

### Polyamine impregnation on MOF/SiO_2_ hybrid materials and bulk MOFs

All the materials were evacuated at 120 °C under vacuum for 1 h. Then, 35 wt% of polyamine-confined sorbents were prepared by impregnating a solution containing 0.54 g of polyamine in 2 mL of CHCl_3_ on 1 g of the evacuated material and were subsequently dried at 120 °C under vacuum for 15 min. Polyamine coordinated sorbents were prepared by impregnating a solution containing 0.54 g of polyamine in 2 mL of dry CHCl_3_ on the evacuated material under N_2_. The mixture was left under N_2_ for 2 h, subsequently washed with dry CHCl_3_ and dried at 80 °C under vacuum for 15 min.

### Polyamines covalently bonded to (Zr)UiO-66(NH_2_)/SiO_2_*via* post-synthesis modification with tris(hydroxymethyl)phosphine

In the first step, 2 g of the previously evacuated sample of 4.2 wt% of (Zr)UiO-66(NH_2_)/SiO_2_ was exposed overnight to the vapors of 200 mg of tris(hydroxymethyl)phosphine (THP) at 100 °C inside a Schlenk flask under vacuum to enhance the degree of amine functionalization (because THP can be easily oxidized when O_2_ is present). The material was thoroughly washed with dry CHCl_3_, and then evacuated at 120 °C under vacuum. Finally, 35 wt% of polyethylenimine (PEI) was introduced into the modified material as previously described for preparing polyamine-confined sorbents.

### CO_2_ adsorption capacity and stability testing of solid sorbents in a PBR

During the next part of the study, 2 g of sorbent were loaded into a PBR and tested for CO_2_ capture under simulated flue gas conditions. Specifically, the conditions were as follows: CO_2_ = 15 v/v%, O_2_ = 4.5 v/v%, H_2_O = 5.6 v/v% in balance with N_2_ at 50 °C during 45 min for the adsorption step, and H_2_O = 5.6 v/v% in balance with N_2_ at 120 °C during 45 min for the regeneration step. To evaluate the stability of the sorbents in the presence of contaminants, controlled concentrations of SO_2_ (50 or 200 ppm), NO_*x*_ (20 ppm of NO and 180 ppm of NO_2_), or H_2_S (1 v/v%) were added during the adsorption step.

### Fluidization and stability test of solid sorbents in an FBR

During the next part of the study, 50 g of the sorbent were loaded into a vFBR. The adsorption was performed by exposing the sorbent to 2 SLPM of 15 v/v% of CO_2_, 8 v/v% of H_2_O in balance with N_2_ at 40 °C until CO_2_ breakthrough was observed and the outlet CO_2_ concentration was stable. Then, between the adsorption and regeneration steps, the reactor was purged with N_2_ to remove the CO_2_ in the gas-phase remaining in the system. Finally, the regeneration step was performed by treating the sorbent with 10 v/v% of H_2_O in N_2_ at 100 °C. This treatment was continued until the CO_2_ was completely desorbed from the sorbent. To evaluate the fluidizability, the sorbents were exposed to 80 v/v% of H_2_O in balance with N_2_ at 100 °C. The pressure drops across the FBR of each sorbent were also examined by varying the N_2_ flow from 0.5 to 4.8 SLPM.

Complete descriptions of the PBR and vFBR, as well as the experimental procedures are included in the ESI† and in our previous work.^18^

## Results and discussion

### The CO_2_ adsorption performance of polyamine-containing MOF/SiO_2_ under simulated flue gas conditions: initial study for (Cr)MIL-101(SO_3_H)/SiO_2_

**Fig. 1:**
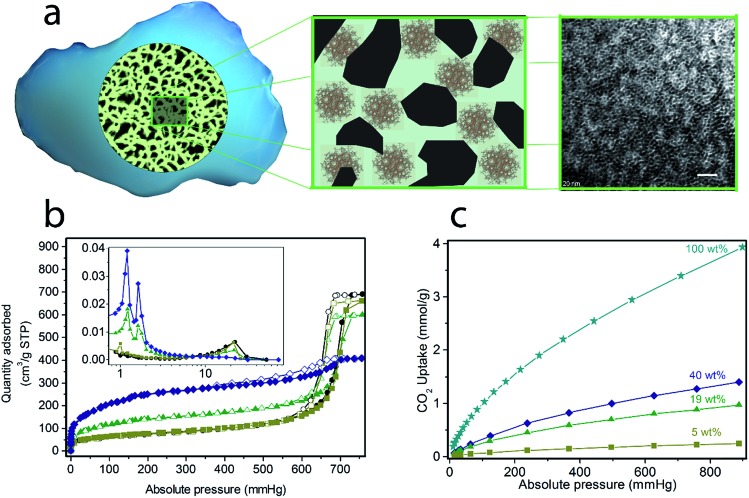
Solid-state synthesis of (Cr)MIL-101(SO_3_H)/SiO_2_ at varying MOF loadings: 5, 19 and 40 wt%. (a) Scheme of the fluidised MOF/SiO_2_ hybrid material showing the dispersion of MOF nanocrystals along mesoporous silica in the transmission electron microscopy image (white scale bar = 20 nm, extended study included in our previous work^19^). N_2_ sorption isotherms at 77 K (b) and CO_2_ sorption isotherms at 273 K (c) for (Cr)MIL-101(SO_3_H)/SiO_2_ at varying MOF loadings: 5 wt% (olive green, ■), 19 wt% (green, ▲), 40 wt% (dark blue, ♦) and 100 wt% (blue, ★). Closed symbols correspond to adsorption branches, whereas open symbols correspond to desorption branches. (inset in b) The pore size distribution was calculated from the Barrett–Joyner–Halenda (BJH) adsorption d*V*/d*D* plot (pore diameter [nm] at *x*-axis and pore volume [cm^3^ g^–1^ nm^–1^] at *y*-axis).

(Cr)MIL-101(SO_3_H) has been selectively grown in a commercially fluidized mesoporous silica (silica[A]) at various loadings, such as 5, 19 and 40 wt%, by following our recently reported procedure.^11^ This MOF was selected as an initial candidate because of its well-known chemical and thermal stabilities and because of the presence of functional groups and available open metal sites for coordinating polyamines, which has been recently reported as an approach for preparing polyamine-containing MOFs for CO_2_ capture.^19^ As shown in Fig. 1, the resulting fluidized MOF/SiO_2_ hybrids exhibited similar physicochemical properties to their bulk counterparts because N_2_ and CO_2_ adsorption was proportional to the MOF loading (measured by X-ray fluorescence [XRF]), thus confirming that these are preserved after integrating MOF nanocrystals within the mesoporous cavities. The presence, loading, and homogeneity of MOF nanocrystals confined on these MOF/SiO_2_ hybrid materials were further confirmed by XRF, X-ray diffraction (XRD), Fourier transform infrared (FTIR), confocal microscopy, transmission electron microscopy (TEM), and scanning electron microscopy (SEM), as described in Fig. 1 and in the ESI.†

**Fig. 2:**
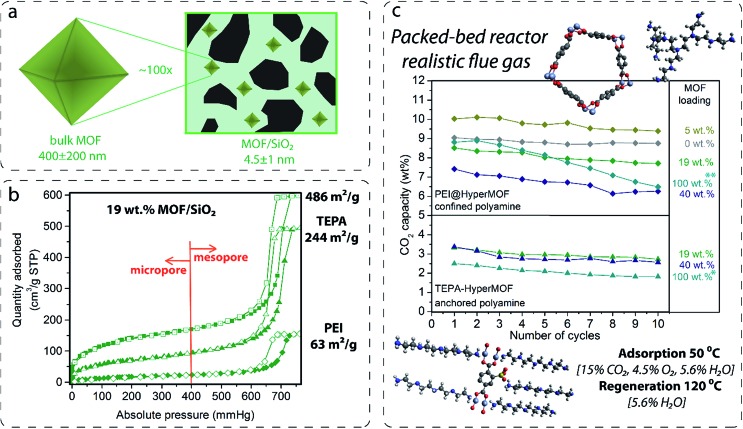
(a) Schematic representation of the particle domain reduction per two orders of magnitude for the bulk (Cr)MIL-101(SO_3_H) crystallites by solid-state synthesis. (b) N_2_ sorption isotherms of evacuated 19 wt% (Cr)MIL-101(SO_3_H)/SiO_2_ containing anchored TEPA (▲) and confined 35 wt% PEI (♦) compared with the empty HyperMOFs (■). (c) CO_2_ adsorption capacity during 10 cycles measured in a packed-bed reactor under simulated flue gas conditions of (Cr)MIL-101(SO_3_H)/SiO_2_ hybrid materials at varying MOF loadings, such as 5 (olive green), 19 (green) and 40 wt% (dark blue), which contained anchored TEPA (▲) or 35 wt% of PEI (♦) confined within the pores. In addition, 100 wt% of MOF loading (blue) corresponds to the bulk MOF impregnated with 35 wt% of TEPA (**) and the bulk MOF material after thoroughly washing it with CHCl_3_ (*) instead of using branched PEI.

Two configurations of polyamine-containing fluidized MOF hybrid sorbents were prepared by using (Cr)MIL-101(SO_3_H)/SiO_2_ hybrid materials: anchored and confined polyamines. The first approach consisted of treating the hybrid materials with linear, but not highly volatile, polyamines, such as tetraethylenepentamine (TEPA) and diethylenetriamine (DETA), in non-coordinating solvents (chloroform) to anchor them *via* both coordinative bonds through the open metal site at the chromium oxocluster and the ionic bond through the free sulfonic acid group at the ligand, as shown in Fig. 2. After washing with chloroform, the resulting composites exhibited a full degree of functionalization for both open metal sites and sulfonic groups, as suggested by the nitrogen to chromium ratios (N : Cr) of *ca.* 10 measured by using a combination of elemental analysis and XRF (see Tables S1 and S2 in the ESI†). TEPA approximately corresponds to one TEPA molecule coordinated to one chromium atom and another reacted with one sulfonic acid group (*i.e.* Cr_3_O[TEPA]_3_[BDC(SO_3_)^–^(TEPA)^+^]_3_). Attempts to coordinate TEPA on bulk (Cr)MIL-101(SO_3_H) under the same conditions resulted in a lower degree of functionalization (N : Cr = 2.8). This finding may be attributed to the constrained diffusion of TEPA molecules through the pore system of the bulk material, especially after the partial blockage of MOF pore apertures by TEPA molecules already anchored. Therefore, MOF nanocrystals dispersed on mesoporous silica exhibiting a 100-times smaller crystal size than their bulk counterparts allow for better polyamine diffusion and coordination along the MOF crystalline domain.

Next, 2 g of these composites were tested in a PBR under simulated flue gas conditions for 10 cycles to evaluate CO_2_ adsorption capacity and stability. As shown in Fig. 2, TEPA coordinated on both 19 and 40 wt% MOF/SiO_2_ exhibited better performance and amine use for CO_2_ capture compared with pure bulk MOFs. 43% of amine utilization was calculated for 19 wt%, 26%, and for 40 wt% and only 14% for bulk MOFs under these experimental conditions, that is, considering that four of the TEPA molecules may coordinate to CO_2_ while one is coordinated to the MOF (Table S2 in the ESI†). Lower CO_2_ adsorption capacity was measured for materials fully functionalized by shorter polyamines because of their lower number of amines per molecule, such as DETA or ethylenediamine (Table S2 in the ESI†). This evidence shows the beneficial effects of having a moderate concentration of well-dispersed MOF nanocrystals anchoring polyamines to enhance the accessibility and efficiency of the amine adsorption sites under simulated flue gas conditions.

**Fig. 3:**
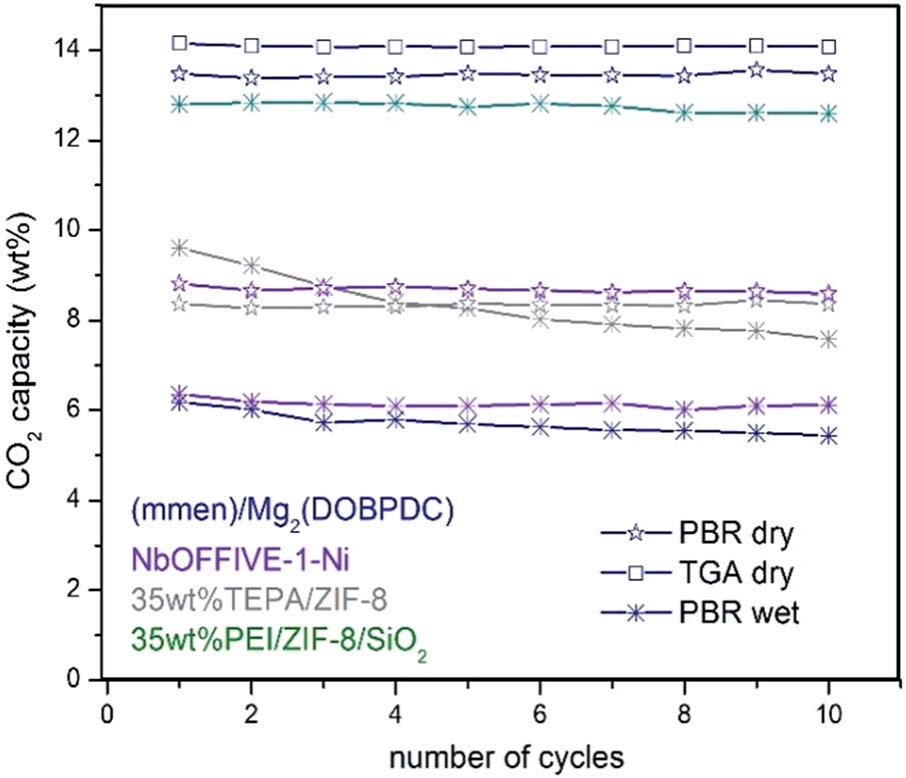
CO_2_ capture of polyamines impregnated on bulk MOFs compared with MOF/SiO_2_ under simulated flue gas conditions in a PBR.

However, the second approach *via* confinement within the pores leads to a higher loading of active polyamines, and thereby higher CO_2_ capacities because the hierarchical mesoporosity and microporosity of the MOF/SiO_2_ hybrid materials can be impregnated with up to 35 wt% of branched polyamines (PEI, *M*_w_ = approximately 800) without revealing signs of stickiness, which would avoid their application in fluidized beds.^18^ The infiltration of PEI on MOF/SiO_2_ microporosity and mesoporosity, as compared in Fig. 2b for 19 wt% of (Cr)MIL-101(SO_3_H)/SiO_2_ containing anchored TEPA (occupying microporosity) and confined 35 wt% of PEI (occupying both microporosity and mesoporosity), has been confirmed by using N_2_ sorption isotherms. Thus, CO_2_ adsorption capacity of confined PEI is also highly dependent on the MOF loading because the presence of 19 and 40 wt% of MOF within the mesoporous silica reduces the CO_2_ adsorption capacity of the amines compared with that of bare silica. This inhibition phenomena may be attributed to the interaction of PEI amines with both open metal sites and free functional groups at the confined MOF and the defective sites located at the MOF boundary inherent to the nanocrystalline domains. Therefore, the CO_2_ adsorption capacity drops proportionally with the MOF loading because a lower capacity was found for 40 wt% MOF loading. Surprisingly, PEI infiltrated on a HyperMOF containing only 5 wt% of MOF leads to a slight enhancement (*ca.* 10 wt%) of the CO_2_ adsorption capacity (Fig. 2c). However, additional experiments confining 35 wt% of TEPA on MOF/SiO_2_ materials revealed a high deactivation because of the leaching of non-coordinated TEPA molecules during the regeneration step at 120 °C in the presence of wet nitrogen. This finding was also observed for 35 wt% of TEPA impregnated on the same bulk MOF and as described later in this manuscript for other impregnated short polyamines (see Fig. 3 and Table 1). Therefore, the use of TEPA as a polyamine was discarded for this application. Branched PEI was found to be more stable for fluidized MOF hybrid sorbents under simulated flue gas conditions.

**Table 1:** Results of the performance for CO_2_ capture in a packed-bed reactor under simulated flue gas conditions for 35 wt% of PEI confined within a selection of MOF/SiO_2_ hybrid materials at varying MOF loadings: CO_2_ adsorption capacity measured for the second cycle and the percentage of deactivation for 10 cycles^*a*^

Entry	MOF	CO_2_^*b*^ (wt%)	Deactivation^*c*^ (%)	MOF^*d*^ (wt%)
1	—	9.0	2.7	0.0
2	(Cr)MIL-101(SO_3_H)	10.0	6.1	4.9
3	(Cr)MIL-101(SO_3_H)	8.3	8.3	19.1
4	(Cr)MIL-101(SO_3_H)	7.1	17.7	40.0
5	(Cr)MIL-101(SO_3_H)	8.8	26.1	100.0
6	(Cr)MIL-101(SO_3_H)^*e*^	5.1	0.1	100.0
7	(Zr)UiO-66(NH_2_)	12.0	2.6	1.5
8	(Zr)UiO-66(NH_2_)	12.5	3.3	4.2
9	(Zr)UiO-66(NH_2_)	10.6	3.4	6.8
10	(Zr)UiO-66(NH_2_)	6.8	5.9	37.6
11	(Zr)UiO-66(NH_2_)	4.6	9.5	100.0
12	**(Zn)ZIF-8**	**12.6**	**1.5**	**4.6**
13	(Zn)ZIF-8	12.5	3.1	7.8
14	(Zn)ZIF-8	10.1	6.5	35.1
15	(Zn)ZIF-8	9.5	15.7	100.0
16	(Zn)ZIF-8^*e*^	8.2	0.9	100.0
17	**(Zn)ZIF-7**	**12.0**	**1.2**	**4.9**
18	Mg_2_(dobpdc)^*f*^	6.2	12.4	100.0
19	Mg_2_(dobpdc)^*e*^ ^,^^*f*^	13.5	0.2	100.0
20	NbOFFIVE-1-Ni^*g*^	6.2	1.1	100.0
21	NbOFFIVE-1-Ni^*e*^ ^,^^*g*^	8.6	2.4	100.0
22	(Co)ZIF-67	12.7	18.8	5.2
23	(Al)MIL-53(NH_2_)	12.2	7.6	3.9
24	(Cr)MIL100	10.4	4.6	5.6
25	(Fe)MIL-100	12.6	24.9	5.3
26	(Zr)PCN-222	11.4	1.4	3.5
27	(Zr)UiO-66	11.9	4.9	4.7
28	(Zr)NU-1000	11.2	7.9	4.2

^*a*^In all cases, 2 g of the solid sorbent were loaded into a PBR blended with SiC_4_ particles as inert with good thermo-conductivity, and 35 wt% TEPA was used for bulk MOFs instead of PEI.

^*b*^CO_2_ adsorption capacity at the second cycle measured in a PBR under simulated flue gas conditions.

^*c*^10 cycle CO_2_ adsorption deactivation.

^*d*^MOF loading calculated by using XRF.

^*e*^Sorbent was measured under dry conditions.

^*f*^32 wt% mmen was used instead of PEI.

^*g*^No polyamine was used.

### Scope for 35 wt% of PEI confined HyperMOFs

We considered to extend this study to other MOF nanocrystals grown within mesoporous silica to evaluate the effects that the MOF composition and loading have on the activity for CO_2_ capture of a defined amount of impregnated PEI. Furthermore, a list of hybrid solid sorbents containing various loadings of a collection of chemically stable MOFs was selected to provide a better understanding about the boost in CO_2_ adsorption capacity observed for moderate MOF loadings. In doing so, MOFs having different metals at the nodes (with both coordinatively unsaturated and saturated sites) and ligands (with and without free functionality) were prepared at various MOF loadings for this scope. To compare our fluidized hybrid materials with the most promising polyamine-containing MOF-based CO_2_ sorbents, bulk Mg_2_(dobpdc) decorated with up to 32 wt% of *N*,*N*′-dimethylethylenediamine (mmen) was prepared according to the literature^16^ and tested under dry (no H_2_O) and simulated flue gas conditions. A similar characterization was conducted for all resulting hybrid solid sorbents listed in Table 1 (also, see the ESI†).

As shown in Table 1, a similar tendency was observed for 35 wt% of PEI impregnated on HyperMOF hybrid materials at various loadings compared with the results presented in Fig. 2c. In all of the cases, a boost in the CO_2_ adsorption capacity was found for *ca.* 5 wt% of MOF loading compared with just PEI impregnated on pure silica, whereas lower CO_2_ adsorption capacity and higher deactivation were measured for higher MOF loadings. Therefore, the initial CO_2_ sorption capacity of PEI amines is improved by the presence of MOF nanocrystals, but at moderate loadings, because a concomitant inhibition mechanism is significantly affecting the adsorption performance for excessive presence of MOFs. The irreversible inhibition pathways may be one or a combination of three scenarios. The first scenario involves neutralization of the PEI amines by defective ligands and/or coordinatively unsaturated metal sites. The second scenario involves oxidation of the PEI amines by MOF metal components at the nodes at high temperatures. The last scenario involves hindrance or aggregation of the PEI amines because of confinement within microporous cavities.

By comparing the results obtained for (Zn)ZIF-8 (entries 12 through 16) or (Zn)ZIF-7 (entry 17), which consists of imidazolate-type ligands, with those of the remainder of the carboxylate-type MOFs, such as (Cr)MIL-101(SO_3_H) (entries 2 through 5) or (Zr)UiO-66(NH_2_) (entries 7 through 11), the initial CO_2_ adsorption capacity and the deactivation rate are less affected by the MOF loading. Thus, boundary crystal defects in the MOF nanocrystals containing carboxylate-type ligands may neutralize more numbers of PEI amines than MOFs consisting of N-type ligands. Therefore, better long-term stability was observed for (Zn)ZIFs compared with that of carboxylate-containing MOFs that exhibit higher deactivation in all cases. However, materials containing Co (entry 22) or Fe (entry 25) at the metal nodes show the highest deactivation rates compared with their isostructural counterpart containing Zn (entry 12) or Cr (entry 24) for the same MOF loadings, respectively. This finding can be attributed to the superior activity of these metals for catalytic amine oxidation.

### Bulk *versus* hybrid MOFs

To the best of our knowledge, literature describing the performance and stability of polyamines confined within bulk MOFs for CO_2_ capture under simulated flue gas conditions in a PBR is very limited. In this current work, such study was included for selected bulk MOFs (Table 1, see entries 5, 6, 11, 15, 16, 18, 19, 20, and 21) to compare the performance with the stability observed for our fluidized MOF/SiO_2_ hybrid sorbents under the same conditions (see Fig. 3). A clear inhibition of the amine capacity was observed for mmen–Mg_2_(dobpdc) when simulated flue gas conditions were used. The presence of 5.6 wt% of H_2_O in the stream leads to a reduction in CO_2_ adsorption capacity from an excellent capacity of *ca.* 14 wt% (entry 19) measured under dry conditions in both thermogravimetric analysis (TGA) and a PBR (confirming reported value of 15.6 wt%) to 6.2 wt% (entry 18) under simulated flue gas conditions. According to elemental analysis, the N contained is also notably reduced from 10.1 wt% (confirming a reported value of 10.3 wt%) to 6.9 wt% (31% N loss) after 10 cycles, probably because of a progressive displacement by the H_2_O molecules of volatile diamines, which are evaporated during the regeneration stage at 120 °C, as also recently described in the literature.^7^

In the same way, deactivation was observed for bulk (Zn)ZIF-8 (entries 15 and 16) and (Cr)MIL-101(SO_3_H) (entries 5 and 6) in the presence of H_2_O, although CO_2_ adsorption capacity is quite similar under both “wet” and dry conditions for (Zn)ZIF-8, but is lower for dry (Cr)MIL-101(SO_3_H) compared with “wet” conditions. In the same way, very low deactivation (*i.e.* <1%) was measured for all polyamine-containing bulk MOFs under dry conditions. Moreover, an FTIR analysis of fresh and used TEPA impregnated in (Cr)MIL-101(SO_3_H) and (Zn)ZIF-8 revealed some differences that may be attributed to the degradation of MOF structures in the presence of polyamines promoted by either O_2_, H_2_O, and temperature or a combination of them (Fig. S6 in the ESI†). In the same way, similar stability issues under simulated flue gas conditions were reported in the literature^20^ for TEPA-impregnated bulk MOFs and were criticized by researchers^21^ for bulk MOFs adsorbing CO_2_ by physisorption, such as (Mg)MOF-74 and SIFSIX-3-Cu, which show a drastic drop in CO_2_ adsorption capacity in a few cycles compared with highly stable copper silicalite. Nevertheless, NbOFFIVE-1-Ni overcame these observed stability issues because of its hydrophobicity (entries 20 and 21), although the CO_2_ adsorption capacity was lower and reversibly dropped by 28% in the presence of 5.6 v/v% of H_2_O (Fig. S8 in the ESI†).

Another hypothetical reason for this inherent drop in CO_2_ adsorption capacity could be caused by the loose polyamine dispersion and subsequent mutual aggregation that leads to an auto-inhibiting effect of the amine sorption sites. This effect was observed for our hybrid materials containing more than 40 wt% of PEI and for PEI confined on pure mesoporous silicas exhibiting both smaller and larger pore sizes (*e.g.* 9.5 nm in SBA-15 or 42 nm in silica[D], respectively, see Fig. S9 in the ESI†) than mesoporous silica(A). In comparison, silica(A) exhibited a optimal polyamine dispersion and was also enhanced by the presence of MOF nanocrystals. In addition, this effect may also be attributed to a hygroscopic inhibition that leads to a reduction of the CO_2_ adsorption capacity because of the accumulation of H_2_O around the highly hydroscopic polyamine-impregnated solids. Therefore, these major limitations for the direct application of polyamine-containing bulk MOFs under simulated flue conditions are efficiently overcome by using a moderate concentration of MOF nanocrystals dispersed within mesoporous silica. Doing so will raise the adsorption efficiency of the impregnated amines while avoiding the inhibition caused by polyamine aggregation and hydroscopic hindrance, which further encourage the use of our MOF/SiO_2_ hybrid solid sorbents for this application.

### Boost in capacity

**Fig. 4:**
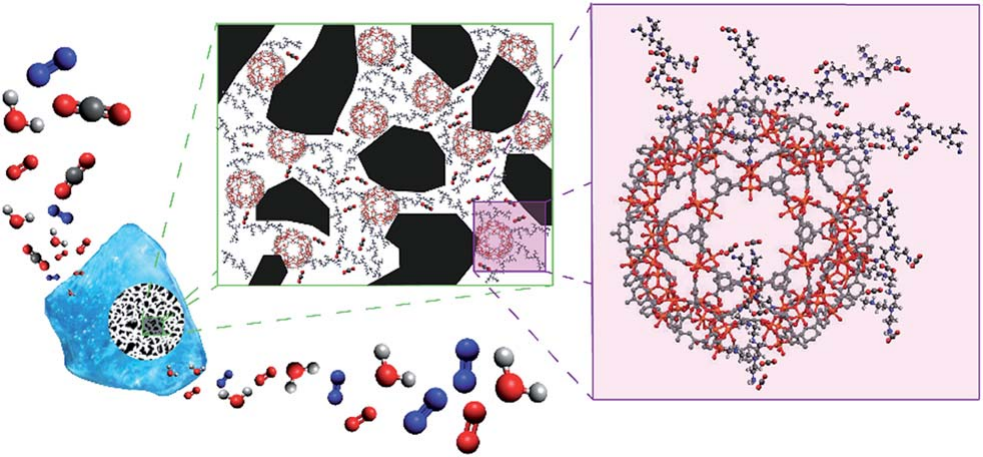
Overview of our novel hybrid CO_2_ solid sorbents.

Several compounds have been used as additives for improving the CO_2_ adsorption capacity of PEI confined on mesoporous silica, such as surfactants,^22^ which enhance the PEI dispersion along the mesoporous cavities, thus active PEI amines are better exposed at the gas–solid interface. Otherwise, a poor dispersion of polyamines commonly involves that a significant number of amines can be sterically hindered by their mutual aggregation by intermolecular H bonds, which inherently leads to the inhibition of their CO_2_ adsorption capacity and the limited CO_2_ diffusion through the amine-impregnated pores. In the same way, well-dispersed MOF nanocrystals may also act as a dispersing agent (see Fig. 4), thus providing a more active surface area to stabilize PEI molecules. MOF nanocrystals within the silica reduce the mesoporosity while those cavities are filled with microporous crystallites, which can interact with PEI molecules by bonding or confining them. Nevertheless, we have determined that the excessive loadings of MOFs lead to the proportional inhibition of the PEI amines. Therefore, an optimal compromise between polyamine dispersion and inhibition was found for *ca.* 5 wt% of MOF loading.

**Fig. 5:**
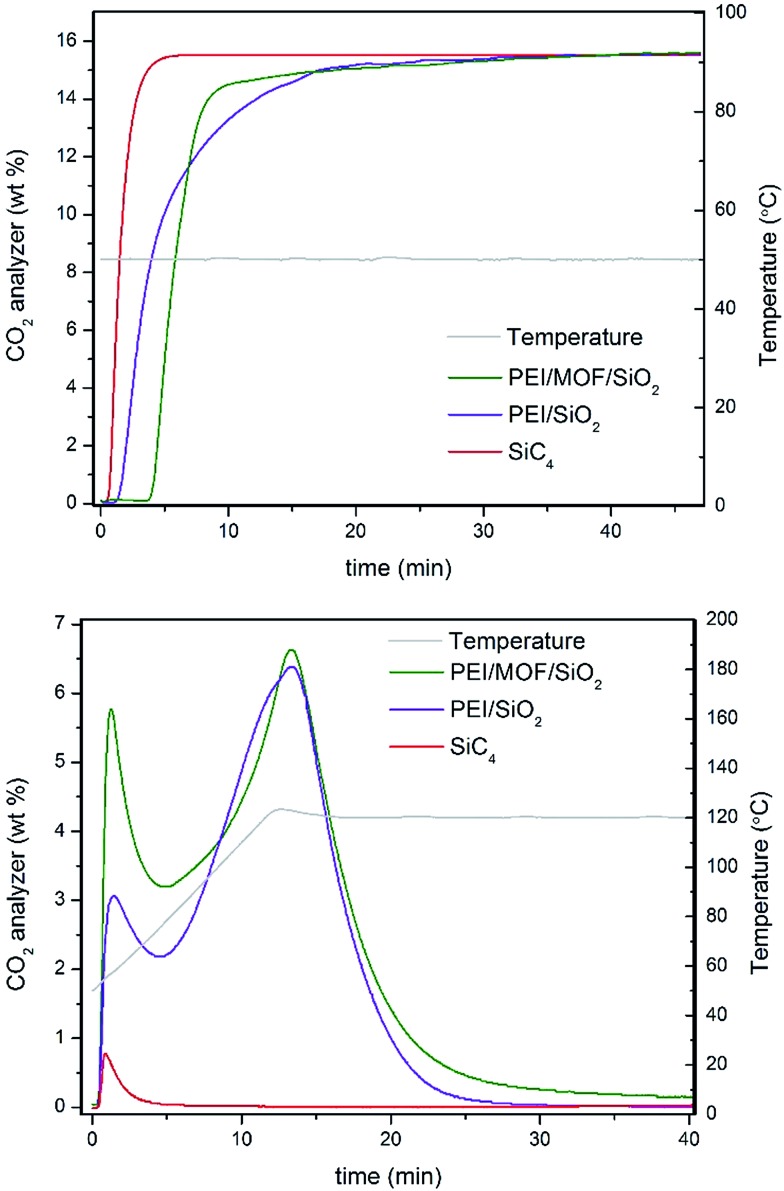
(Top panel) Adsorption and (Bottom panel) regeneration profiles of 35 wt% of PEI/4.6 wt% of (Zn)ZIF-8/SiO_2_ (entry 12, Table 1) and 35 wt% of PEI/SiO_2_ (entry 1, Table 1).

To provide insight into the influence the presence of MOF nanocrystals has on the CO_2_ dynamics of impregnated PEI, the adsorption–desorption profiles for 35 wt% of PEI confined on 4.6 wt% of (Zn)ZIF-8/SiO_2_ and bare SiO_2_ at the tenth cycle are compared in Fig. 5. The adsorption breakthrough profile for the MOF-containing solid sorbent shows a superior CO_2_ adsorption to PEI/SiO_2_, as shown in Table 1. The characteristic adsorption–regeneration profiles for the PBR filled with inert SiC_4_ are represented by the red line, which determines the dead volume of the PBR. However, the regeneration profile shows an interesting difference in terms of CO_2_ desorption for the composite incorporating MOF nanocrystals. Larger concentrations of the early released adsorbate are measured for the MOF/SiO_2_ hybrid sorbent, which is attributed to weakly adsorbed CO_2_*via* physisorption because the temperature required to release them is lower than 80 °C.

PEI/SiO_2_ also exhibited an early release, but it is mainly attributed to CO_2_ trapped in the dead volume of the PBR, as suggested by comparison with the profile for the inert SiC_4_. In addition, PEI/MOF/SiO_2_ exhibited higher CO_2_ desorption between 80 and 100 °C, which suggests the slightly better use of the PEI amines for CO_2_ chemisorption as well. This result highlights the unusual dual adsorption performance of our hybrid sorbents containing MOF nanocrystals compared with their pure silica counterpart.

### Long-term stability in the presence of SO_2_ and NO_*x*_ contaminants in a PBR

**Fig. 6:**
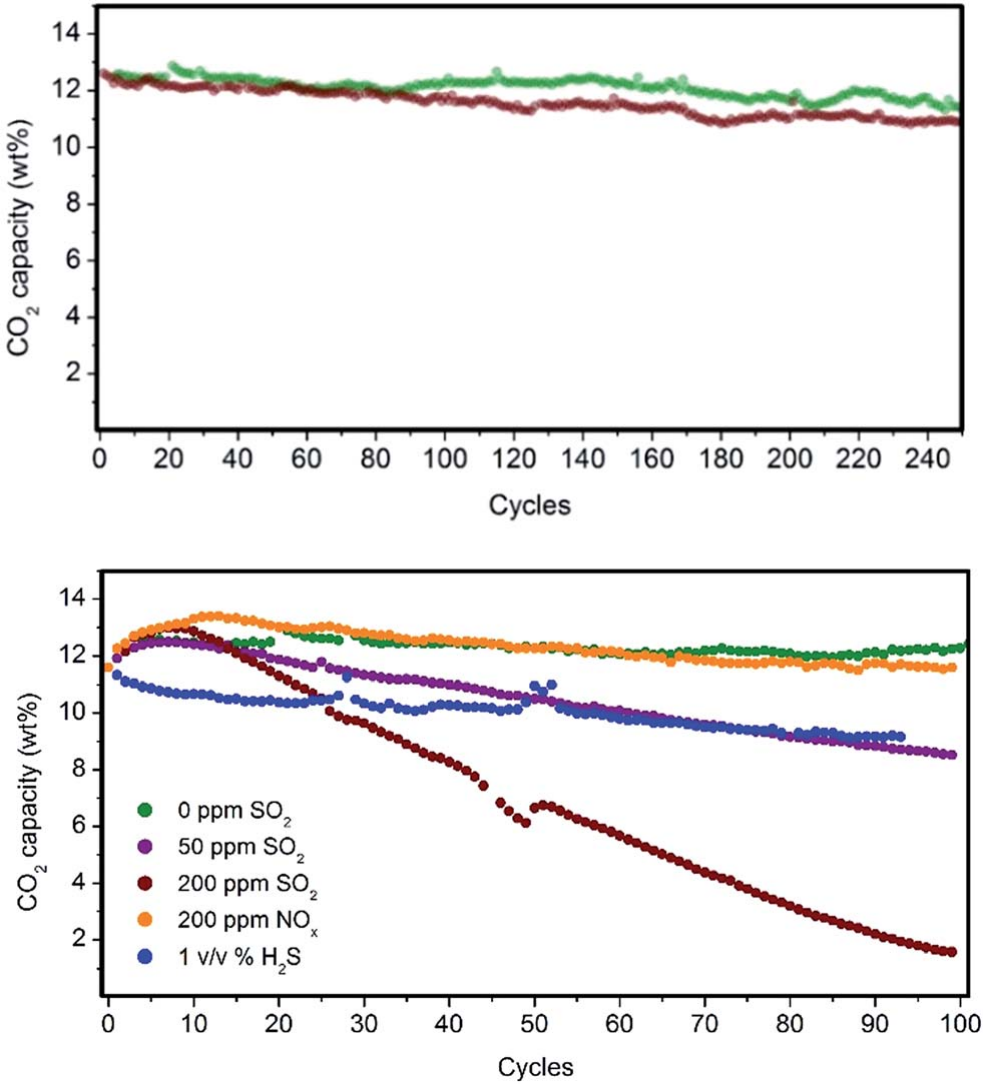
(Top panel) Long-term CO_2_ adsorption performance stability of PEI/(Zn)ZIF-8/SiO_2_ (green) and PEI/(Zn)ZIF-7/SiO_2_ (dark red) under simulated flue gas conditions. (Bottom panel) Long-term CO_2_ adsorption performance stability of PEI/(Zn)ZIF-8/SiO_2_ under the presence of SO_2_, NO_*x*_ and H_2_S contaminants.

Fluidized hybrid sorbents containing zinc imidazolates, such as (Zn)ZIF-8 and (Zn)ZIF-7, have demonstrated good CO_2_ adsorption capacity under simulated flue gas conditions because they exhibited 140% higher CO_2_ adsorption capacity than the reference PEI impregnated on bare mesoporous silica (Table 1, entry 1) and the lowest 10-cycle deactivation (Table 1, entries 12 and 17) compared with other MOFs. This finding encourages us to analyse these two sorbents for long-term stability in a PBR. Both sorbents exhibited excellent stability for 250 cycles under simulated flue gas conditions, as shown in Fig. 6 and resumed in Table S3 in the ESI.†

Flue gas from coal-fired power plants typically contains other acid-gas impurities, such as SO_2_ and NO_*x*_, that can dramatically influence the CO_2_ capture efficiency.^23^,^24^ Therefore, during this current work, the performance of our MOF-based sorbents in the presence of these contaminants was evaluated. The results show a clear deactivation of the CO_2_ adsorption capacity of the sorbents in the presence of SO_2_. This deactivation is because of the irreversible reaction occurring during the adsorption step between SO_2_ and PEI amines, which are not further active for the CO_2_ capture (Fig. 6).

Amine deactivation was proportional to the SO_2_ concentration, as shown by the S content determined by CHNS elemental analysis after 100 cycles (see Table S4 in the ESI†). Also, FTIR spectra of these samples measured after 100 cycles revealed the clear, irreversible binding of SO_2_ to the hybrid sorbent (Fig. S7 in the ESI†). However, excellent stability at higher NO_*x*_ concentrations was observed. Therefore, MOF nanocrystals within the hybrid solid sorbent did not reduce the tendency of PEI amines to be deactivated by irreversible binding with SO_2_, as a similar deactivation for PEI/SiO_2_ was observed by our group.^18^ Therefore, another unit should be added upstream for scrubbing the SO_2_ levels in the flue gas down to a single-digit parts per million level before reaching the FBR to elongate the life of the hybrid solid sorbents and reduce the make-up rate. Good stability was measured in the presence of H_2_S, typically found in sour gas streams, although an initial drop in capacity was observed because the study was performed under dry conditions and without O_2_ because H_2_ was in the H_2_S cylinder.

### FBR

The fluidizability of the PEI-impregnated MOF/SiO_2_ solid sorbents and their performance under realistic conditions are of high importance to minimize unexpected operational issues to further scale-up this technology. In this work, we report the first example of CO_2_ capture from simulated flue gas in a fluidized bed configuration by using a MOF-based CO_2_ solid sorbent. Application of these materials in this configuration has never been proposed because of poor attrition and handling, as well as a lack of fluidizability of bulk MOFs. In doing so, we have developed an experimental setup consisting of a glass-type column. The column works as an absorber and as a regenerator, where the fluidization of the sorbent is carried out with a gas stream^25^ (see the ESI†), which permits the visual evaluation of the sorbent fluidizability.

**Fig. 7:**
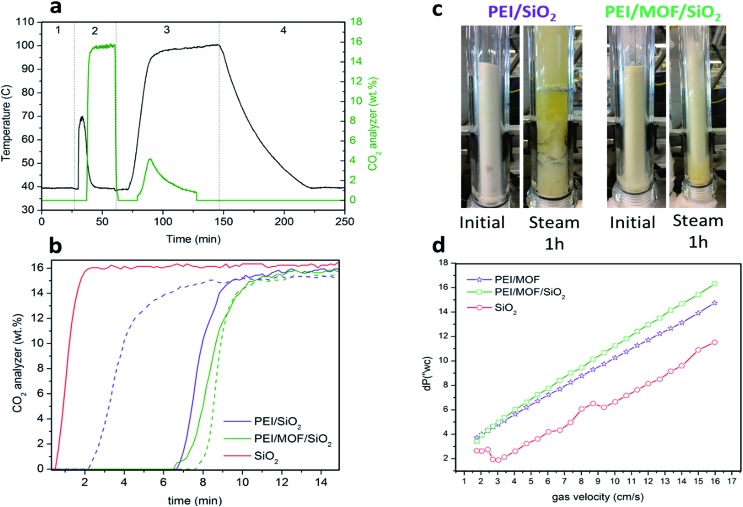
Performance of PEI/MOF/SiO_2_ sorbents for CO_2_ capture in a fluidized bed reactor (FBR) under simulated flue gas conditions compared with conventional that of PEI/SiO_2_ sorbents. (a) Example of the adsorption–regeneration profile for PEI/MOF/SiO_2_ in the FBR: 1-purge (10% of H_2_O/N_2_), 2-adsorption (15% of CO_2_/10% of H_2_O/N_2_), 3-regeneration (10% of H_2_O/N_2_) and 4-purge/cooling (10% of H_2_O/N_2_). (b) Breakthrough adsorption curves. The solid line represents a fresh sorbent and dashed line represents a sorbent exposed to a steam for 1 h. The sorbents were dried with 2 SLPM of N_2_ at 100 °C for 2 h before the second adsorption–regeneration cycle. (c) A photograph of the sorbents before (on left) and after (on right) steam treatment for 1 h. (d) Pressure drop *versus* gas velocity (cm s^–1^).

During this study, the PEI/SiO_2_ sorbent and the most promising PEI/MOF/SiO_2_ sorbent (*i.e.* 4.6 wt% of [Zn]ZIF-8/SiO_2_) were exposed to a series of testing conditions to evaluate their fluidizability. First, the sorbents were subjected to an adsorption–regeneration cycle to determine their CO_2_ adsorption breakthrough curves. Fig. 7b shows the CO_2_ breakthrough curve for both sorbents during the adsorption step compared with that of bare SiO_2_, which allows the dead volume of the system to be determined.

The breakthrough for the bare SiO_2_ occurred after approximately 30 s, whereas the use of 50 g of the solid sorbent containing PEI resulted in a significantly larger lag-time in breakthrough (*ca.* 6 to 7 min), suggesting that both sorbents are capable of deep CO_2_ scrubbing, close to 100% efficiency, before saturation. As observed in the results obtained in the PBR (Table 1), the sorbent containing MOF nanocrystals also exhibited higher adsorption capacity than PEI/SiO_2_ in the vFBR.

Afterwards, both sorbents were exposed to a stream containing 80 v/v% of H_2_O balanced with N_2_ at 100 °C for 1 h. These very aggressive conditions in the regeneration step were used because the stability under these conditions is crucial for further application, as the CO_2_ adsorption capacity of the sorbent could significantly drop because of the amine leaching under steam stripping. It is important to note that the extremely long exposure time of 1 h used for this experiment does not represent the actual residence times of the fluidized sorbent in the regenerator, which is typically 2 to 5 min. Fig. 7c is a photograph of the sorbent bed during the initial CO_2_ loading test and after exposure to steam stripping for 1 h. Surprisingly, PEI/SiO_2_ showed a more severe amine leaching in comparison with the material containing MOF nanocrystals under these conditions. Moreover, the PEI/SiO_2_ sorbent became yellow and wet and stuck to the internal reactor wall, completely losing its fluidizability. In contrast, the MOF-containing sorbent appeared to be more resilient to the steam stripping because the material maintained its fluidizability over 1 h of testing. These results are consistent with the breakthrough adsorption curves measured for these two sorbents after the steam exposure.

After a drying treatment with 1 SLPM of N_2_ at 100 °C for 2 h, CO_2_ adsorption capacity was practically maintained for PEI/MOF/SiO_2_, whereas a significant drop was observed for the MOF-free sorbent (see Fig. 7b). This improved steam stability of the sorbents containing ZIF-8 nanocrystals can be attributed to the well-known hydrophobicity of the MOFs, which can be enhanced further by using longer aliphatic substituents of the imidazole ring.^26^ Currently, the influence on the CO_2_ capture performance of other substituents at the imidazole ring of PEI/MOF/SiO_2_ sorbents is under investigation by our group.

Finally, the pressure drops across the FBR reactor bed for all the materials was also examined by varying the N_2_ flow from 0.5 to 4.8 L min^–1^ (SLPM). The sorbent containing MOF nanocrystals exhibited a slightly higher pressure drop than PEI/SiO_2_, which can be attributed to the higher density because of the presence of the MOF (0.75 kg m^–3^ for PEI/MOF/SiO_2_*versus* 0.68 kg m^–3^ for PEI/SiO_2_). The main benefits of using denser fluidized sorbents are the reduction of the column size, the increment of the gas velocity and the lower sorbent carried over by flue gas. Nevertheless, an excessive pressure drop in the system can lead to a higher energy requirement to drive the fluidized sorbent through the bed. Fortunately, the sorbents containing moderate MOF loadings demonstrate an equilibrated combination of opposite features in terms of fluidizability when operating at the optimal gas velocity range (0.1–0.2 m s^–1^ (ref. 27)) (*i.e.* denser particles, but not involving drastic pressure drops).

### Scale-up and cost analysis study

During this work, we also demonstrated the scale-up of the approach for preparing our novel fluidized hybrid sorbents because up to 1 kg of material was prepared by following our recent published solid-state synthesis approach^11^ (photographs of the big batch are in Fig. S10 in the ESI†). In addition, the use of inexpensive chemicals makes the synthesis very cost effective compared with the costly bulk MOF consisting of expensive ligands and polyamines, such as mmen used for Mg_2_(dobpdc).

The price determined in our group for the kilogram-scale preparation of these sorbents is approximately $20 per kg according to the cost evaluation study we recently conducted (Fig. S11 in the ESI†). This cost can be reduced to $15 per kg by implementing some cost-saving measures such as recycling solvents and finding alternative mesoporous silica and PEI vendors. That reduced cost is in the same range as the production cost for some limited bulk MOFs *via* liquid assisted grinding (LAG), as recently reported in the literature.^28^

### Future directions toward more stable fluidized solid sorbents: polyamines covalently bonded to MOF/SiO_2_ hybrid materials

Using pure steam for the regeneration stage is desirable for intensifying the CO_2_ capture process from post-combustion flue gas because H_2_O can be easily condensed, leading to a pure CO_2_ stream. Nevertheless, steam can progressively displace or wash out active polyamines from current solid sorbents based on PEI-impregnated fluidized sorbents. This limitation may also affect the long-term stability of our novel hybrid solid sorbents during the regeneration stage in an FBR using pure steam because of the relatively weak interactions between MOF nanocrystals and polyamines because H_2_O molecules can displace coordinated amines as previously discussed for mmen–Mg_2_(dobpdc). Therefore, an efficient approach for covalent bonding of polyamines to fluidized solids is a high priority.

**Fig. 8:**
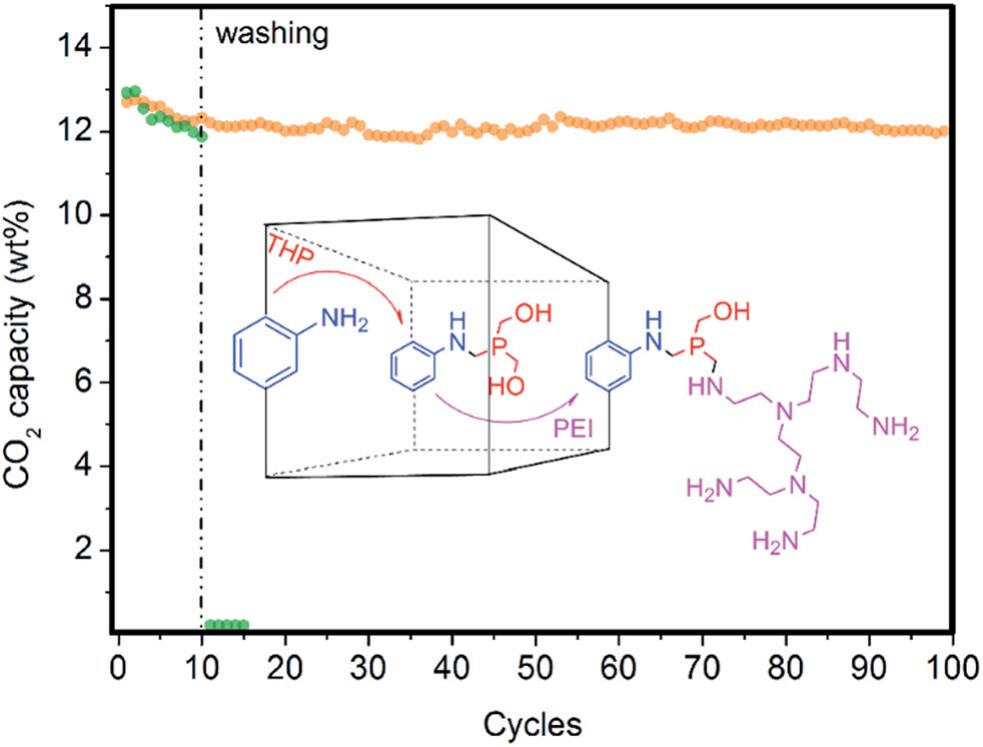
Results of the CO_2_ performance and stability test of 35 wt% of PEI/4.6 wt% of (Zr)UiO-66(NH_2_)/SiO_2_ (green) and 35 wt% of PEI/THP/4.6 wt% of (Zr)UiO-66(NH_2_)/SiO_2_ (orange) in a packed-bed reactor (PBR) under simulated flue gas conditions. Sorbents were thoroughly washed *ex situ* with MeOH after the tenth cycle and were re-loaded into the PBR. The scheme shows the post-synthesis modification of (Zr)UiO-66(NH_2_) nanocrystals confined on SiO_2_*via* two steps: gas-phase treatment with THP followed by PEI impregnation.

During the past decade, a wide collection of strategies for post-synthesis modification have been developed for MOFs containing free functional groups at the organic ligand, especially for amino functionality at the aminoterephthalate ligand.^24^ However, to the best of our knowledge, an efficient, one-pot, environmentally friendly and general approach for covalently bridging amine to amine is still missing in MOF chemistry. However, the use of THP as a coupling reagent for the immobilization of enzymes onto chitosan films *via* Mannich-type condensation resulted in greater enzyme performance than many conventional coupling routes involving glutaraldehyde.^23^,^29^ Hence, we applied this efficient approach of creating strong covalent bonds amine-to-amine *via* a methylene phosphine bridge or bridges (N–P–N) to link PEI to (Zr)UiO-66(NH_2_) nanocrystals while keeping all of the resulting amines still active for CO_2_ adsorption. This approach is in contrast to conventional glutaraldehyde that forms weak imine bonds, which do not exhibit CO_2_ chemisorption (see Fig. 8).

An optimal phosphine grafting (99% by XRF) was achieved *via* gas-phase phosphine functionalization and the material retained most of the PEI impregnated (95% by elemental analysis) after thoroughly washing the material with methanol after 10 cycles, as shown in Fig. 8. This approach provides a powerful synthetic tool to bind amine-containing organic or organometallic compounds to amine-containing MOFs or other solid supports, preserving their activity to be subsequently used for a variety of applications. The applications range from gas adsorption or separation to heterogeneous catalysis to bioimaging or drug delivery.

## Conclusions

MOFs have been successfully engineered into a fluidized form *via* solid-state synthesis within mesoporous silica, demonstrating excellent fluidizability and handling and improved attrition resistance. MOF/SiO_2_ hybrid materials containing a moderate loading of Zn imidazolate MOFs (*ca.* 5 wt%) have been found to be optimal to boost the performance of the 35 wt% – impregnated polyamines for CO_2_ capture under simulated flue gas conditions because CO_2_ adsorption capacity and stability are progressively inhibited by increasing the concentration of MOF nanocrystals.

Long-term stability tests in the presence of contaminants and experiments on a visual FBR showed their excellent stability, fluidizability, and outstanding performance under steam regeneration because of the hydrophobicity conferred by the presence of MOF nanocrystals.

## Conflicts of interest

There are no conflicts to declare.

## Supplementary Material

Supplementary information
