# Supplementary material for: Flying MOFs: polyamine-containing fluidized MOF/SiO2 hybrid materials for CO2 capture from post-combustion flue gas
Source: Chem Sci. 2018 Apr 11;9(20):4589–99. doi: 10.1039/c7sc05372j (PMC5969507; doi:10.1039/c7sc05372j)
Supplement: Supplementary file 1 [file SC-009-C7SC05372J-s001.pdf]

Electronic Supplementary Material (ESI) for Chemical Science.
This journal is © The Royal Society of Chemistry 2018

Flying MOFs: Polyamine-containing fluidized MOF/SiO_2 hybrid materials for CO_2 capture from post-combustion flue gas

Ignacio Luz, Mustapha Soukri*, and Marty Lail

RTI International, Post Office Box 12194, Research Triangle Park, NC 27709-2194.

Supporting information

Experimental and general information

Chemicals.

All chemicals were used as received from Sigma-Aldrich without further purification. Triethylamine (TEA), *N*,*N*-dimethylformamide (DMF), chloroform ($CHCl_3$) and methanol (MeOH), branched polyethyleneimine (PEI, M_w ~ 800), tetraethylenepentamine (TEPA), diethyelenetriamine (DETA), N,N'-dimethylethylenediamine (mmen), trishydroxyphosphine (THP, 90%), $Cr(NO_3)_3 \cdot 9H_2O$, $FeCl_3 \cdot 6H_2O$ $Al(NO_3)_3 \cdot 9H_2O$, $Co(NO_3)_2 \cdot 6H_2O$, $ZrOCl_2 \cdot 8H_2O$, $Zn(NO_3)_2 \cdot 9H_2O$, $Fe(NO_3)_3 \cdot 9H_2O$, $Mg(NO_3)_2 \cdot 6H_2O$, 1,4-benzenedicarboxylic acid (H_2BDC), 1,3,5-benzenetricarboxylic acid (H_3BTC), 2-aminoterephthalic acid ($H_2BDC(NH_2)$), monosodium 2-sulfoterephthalate ($H_2BDC(SO_3Na)$), 2,5-dihydroxyterephthalic acid (H_4DOBDC), 2-methylimidazole (HMeIM), benzimidazole (HBnIM) and tetrakis(4-carboxyphenyl)-porphyrin (H_4TCPP). 1,3,6,8-tetrakis(p-benzoic acid)pyrene (H_4TBAPy) [1] and 4,4'-dihydroxibiphenyl-3,3'-dicarboxylic acid ($H_4DOBPDC$)[2] were synthetized according to the published procedure. Mesoporous silica(A) [75-250 µm] was kindly supplied by our commercial partner (physicochemical properties are reported in our previous work[3]). Mesoporous silica was degassed at 120 °C overnight under vacuum to remove the adsorbed water prior to use.

Ligand salt precursors. Na_2BDC and Na_3BTC ligand salt precursors were prepared from their acid form in water with the stoichiometric amount of NaOH necessary to deprotonate the carboxylic acid of the organic linker followed by a purification step via precipitation in acetone. Alternatively, ligand salt precursor solutions for $H_2BDC(NH_2)$ and H_4TCPP were directly prepared with the stoichiometric amount of TEA, thereby skipping the step of isolating the ligand salt. $H_2BDC(SO_3Na)$ and HMeIM were directly dissolved in water.

Characterization techniques.

Scanning electron microscopy (SEM) images were acquired on a FEI Quanta 200 FEG Analytical Scanning Electron Microscope using a beam energy of 15 kV. **Transmission electron microscopy (TEM)** experiments were performed in a Hitachi 7000 100 kV with AMT digital camera. **N_2 sorption isotherms** were measured in a Micromeritics ASAP (Accelerated Surface Area and Porosimetry) 2020 System. Samples were weighted into tubes with seal frits and degassed under vacuum (<500 µm Hg) with heating. They were initially heated at 150 °C and held for 4 hours, and finally cooled to room temperature and backfilled with N_2. The samples were re-weighted before analysis. The analysis adsorptive was N_2 at 77K. A multi-point BET surface area was determined from 6 measurements at relative pressures (P/Po) ranging from 0.050 to

0.300 satisfying the four criteria suggested by Rouquerol[4]. Single point adsorption total pore volume was measured near saturation pressure (Po ≈ 770 mmHg). Adsorption average pore width was also calculated. Pore size distribution plot was determined by BJH method with Halsey thickness curve equation and Faas BJH correction. **X-ray Fluorescence (XRF)** analysis were performed in a ARL Thermo Scientific (Ecublens, Switzerland) Perform'X Wavelength-Dispersive X-ray Fluorescence (WDXRF) equipped with an X-ray tube 5GN-type Rh target with ultra-thin 30 μm Be window to maximize light element response**.** 4000 W power supply for 60 kV max or 120 mA max with two detectors (flow proportional and scintillation) and seven analyzer crystals to achieve a broad elemental range**. X-ray diffraction (XRD)** patterns were recorded using a Panalytical Empyrean X-ray diffractometer with Cu Kα radiation (λ=1.54778 Å). **Attenuated total reflection (ATR) FTIR** spectroscopy measurements were performed in the range of 4000–400 cm^{-1} with a Perkin Elmer Spectrum 100 FTIR spectrometer.

Solid state synthesis of MOF/SiO_2 hybrid materials

19 wt.% (Cr)MIL-101(SO_3H)/SiO_2 (see HyperMOF-A2a in reference[3])

1st) Ligand impregnation: 10 mL of an aqueous solution containing 2 g $H_2BDC(SO_3Na)$ was impregnated to 5 grams of evacuated mesoporous silica(A) and was dried at 50 °C under vacuum in a rotavapor for 2 h.

2nd) Ligand acidification: the resulting dry material [$H_2BDC(SO_3Na)$/Silica(A)] was placed in a tubular calcination reactor where was first treated with a nitrogen flow saturated with concentrated HCl (37%) for 2 hours at room temperature and after purged with a nitrogen flow for 2 h to remove the excess of HCl.

3rd) Metal impregnation: 7.5 mL of an aqueous solution containing 1.5 gr of $Cr(NO_3)_3·9H_2O$ in 7.5 mL of H_2O was impregnated to the compound [$H_2BDC(SO_3H)$/Silica(A)]. The resulting solid [$Cr(NO_3)_3$/$H_2BDC(SO_3H)$/Silica(A)] was finally dried at 50 °C under high vacuum in a rotavapor for 2 h. All the impregnation steps were done via incipient wetness impregnation.

4th) Synthesis conditions and washing: The solid [$Cr(NO_3)_3$/$H_2BDC(SO_3H)$/Silica(A)] was separated in two 125 mL stainless steel Parr autoclave (> 40 % void space) at 190 °C for 24 h after adjusting the water contain of the solid to 15-20 wt.%. After cooling the autoclave, the resulting products were thoroughly washed with distillated water in a filtration funnel.

5th) Washing treatment and activation: the material was washed overnight in a Soxhlet with MeOH. All the materials were activated overnight at 120 °C under vacuum.

A second solid state synthesis was carried out by using 19 wt.% (Cr)MIL-101(SO_3H)/SiO_2 to obtain 40 wt.% (Cr)MIL-101(SO_3H)/SiO_2. To prepare 4.9 wt.% (Cr)MIL-101(SO_3H)/SiO_2, 0.5 g of $H_2BDC(SO_3Na)$ and 0.35 gr of $Cr(NO_3)_3·9H_2O$ were used instead. Same procedure was followed for preparing 5.6 wt.% (Cr)MIL-100/SiO_2, 0.2 gr of H_3BTC and 0.35 gr of $CrCl_3·6H_2O$ were used instead.

30 wt.% (Zr)UiO-66(NH_2) (see HyperMOF-E2a in reference[3])

Solid state synthesis was followed to obtain 37.6 wt.% (Zr)UiO-66(NH_2) with the following variations: 1st) 1.5 gr of $H_2BDC(NH_2)$, 2.35 mL of TEA and 10 mL H_2O; 2nd) conventional acidification step; 3rd) 2.5 gr of $ZrOCl_2·8H_2O$ in 7.5 mL of H_2O; 4th) 15-20 wt.% DMF and 120 °C for 12h; and 5th) conventional washing and activation treatment.

Moderate concentrations of (Zr)UiO-66(NH_2) on SiO_2 were obtained via incipient wetness impregnation in one step of metal and ligand mixture in DMF as following: 20, 40 and 80 mg of $H_2BDC(NH_2)$, and 50, 100 and 200 mg of $ZrOCl_2 \cdot 8H_2O$ in 2 mL of DMF per gram of SiO_2 for 1.5, 4.2, and 6.8 wt.% MOF loadings, respectively. The materials also were heated at 120 °C for 12h and conventionally washed/activated. Same procedure was followed for preparing 4.7 wt.% (Zr)UiO-66/SiO_2 (40 mg of $H_2(BDC)$ and 100 of $ZrOCl_2 \cdot 8H_2O$ in 2 mL of DMF per gram of SiO_2), 3.5 wt.% (Zr)PCN-222/SiO_2 (50 mg of H_4TCPP and 100 of $ZrOCl_2 \cdot 8H_2O$ in 2 mL of DMF per gram of SiO_2), 4.2 wt.% (Zr)NU-1000/SiO_2 (50 mg of H_4TBAPy and 100 of $ZrOCl_2 \cdot 8H_2O$ in 2 mL of DMF per gram of SiO_2), 3.9 wt.% (Al)MIL-53(NH_2)/SiO_2 (40 mg of $H_2BDC(NH_2)$ and 100 of $Al(NO_3)_3 \cdot 9H_2O$ in 2 mL of DMF per gram of SiO_2), 5.3 wt.% (Fe)MIL-100/SiO_2 (40 mg of H_3BTC and 100 of $Fe(NO_3)_3 \cdot 9H_2O$ in 2 mL of DMF per gram of SiO_2),

35.1 wt.% (Zn)ZIF-8 (see HyperMOF-G1a in reference[3])

Solid state synthesis was followed to obtain 35.1 wt.% *(Zn)ZIF-8* with the following variations: 1st) 2.4 gr of $Zn(NO_3)_3 \cdot 9H_2O$ in 10 mL of MeOH; 2nd) no acidification step; 3rd) 1.8 gr of HmeIM in 7.5 mL MeOH; 4th) TEA vapor treatment; and 5th) conventional washing and activation treatment.

Moderate concentrations of *(Zn)ZIF-8* on SiO_2 were obtained via incipient wetness impregnation in one step of metal and ligand mixture in MeOH as following: 60 and 120 mg of HmeIM, and 80 and 160 mg of $Zn(NO_3)_2 \cdot 9H_2O$ in 2 mL of MeOH per gram of SiO_2 for 4.6 and 7.8 wt.% MOF loadings, respectively. The materials were also treated with TEA vapor and conventionally washed/activated. Same procedure was followed for preparing 4.9 wt.% (Zn)ZIF-7/SiO_2 (80 mg of HmeIM and 80 of $Zn(NO_3)_3 \cdot 9H_2O$ in 2 mL of MeOH per gram of SiO_2) and 5.2 wt.% (Co)ZIF-67/SiO_2 (140 mg of HBnIM and 80 of $Zn(NO_3)_2 \cdot 9H_2O$ in 2 mL of MeOH per gram of SiO_2).

Packed-bed reactor

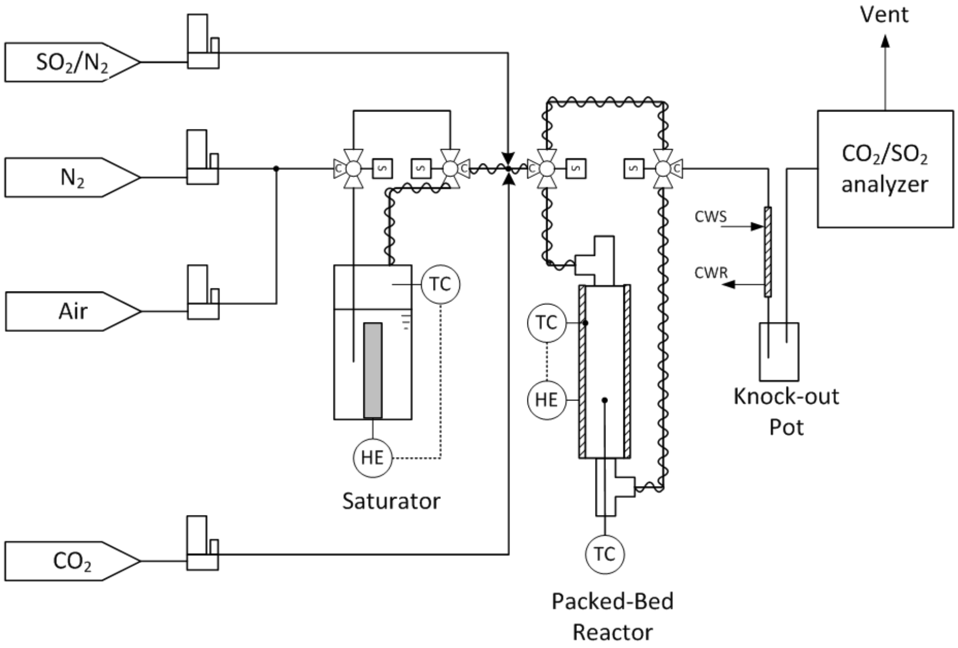

Scheme S1. Process flow diagram of the RTI's packed bed reactor.

A laboratory-based automated packed-bed reactor (PBR) system (developed by RTI) was used to evaluate the sorbent's CO_2 capture performance by executing multicycle CO_2 absorption/regeneration test conditions with minimal operator interaction. As shown in Scheme 1, the PBR system consists of three main sections: (1) feed gas generation, (2) packed-bed reactor, and (3) gas analysis. The feed gas generation system consists of a bank of electronic gas mass flow controllers and a temperature-controlled water saturator. Water content in the gas stream leaving the saturator is controlled and adjusted by increasing or decreasing the saturator temperature. This arrangement allows for the generation of a wide range of feed gas compositions, including wet and dry gas mixtures. All lines downstream of the saturator are heat traced to avoid H_2O condensation and to preheat the gas to the reactor conditions. The gas then enters the top of the packed-bed reactor, flows downward through the sorbent bed, and exits the reactor bottom. The packed-bed reactor is a 0.5 in. OD, 8 in. tall stainless-steel tube with temperature control enabled by well-tuned band heaters. A thermocouple is situated in the middle of the sorbent bed to measure the sorbent bed temperature during absorption and regeneration. The reactor effluent is cooled to 10–12°C in a tube-in-tube heat exchanger to remove excess water which is collected in a knock-out pot. Exiting the knock-out pot is a gas stream containing

1–2 vol% H_2O. The CO_2 and SO_2 concentrations in the reactor effluent were continuously monitored using a Horiba VA-3000 analyzer. Two pairs of solenoid switching valves are used to direct the gas flow through or bypass the reactor and saturator, allowing the PBR to operate highly flexible experimental procedures. Process measurements are taken and control achieved using a RTI-developed data acquisition and process control system.

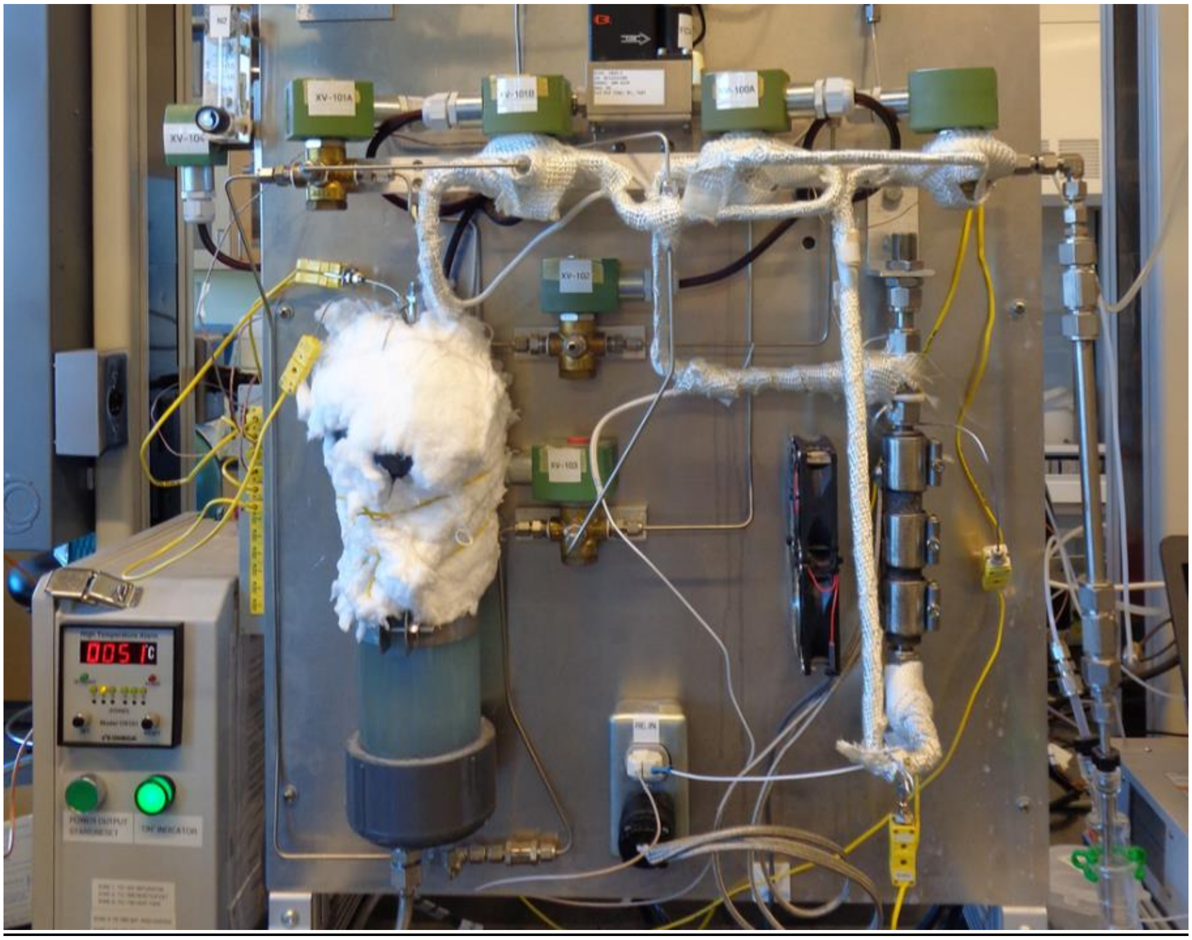

Figure S1. Image of the PBR.

Fluidized-bed reactor

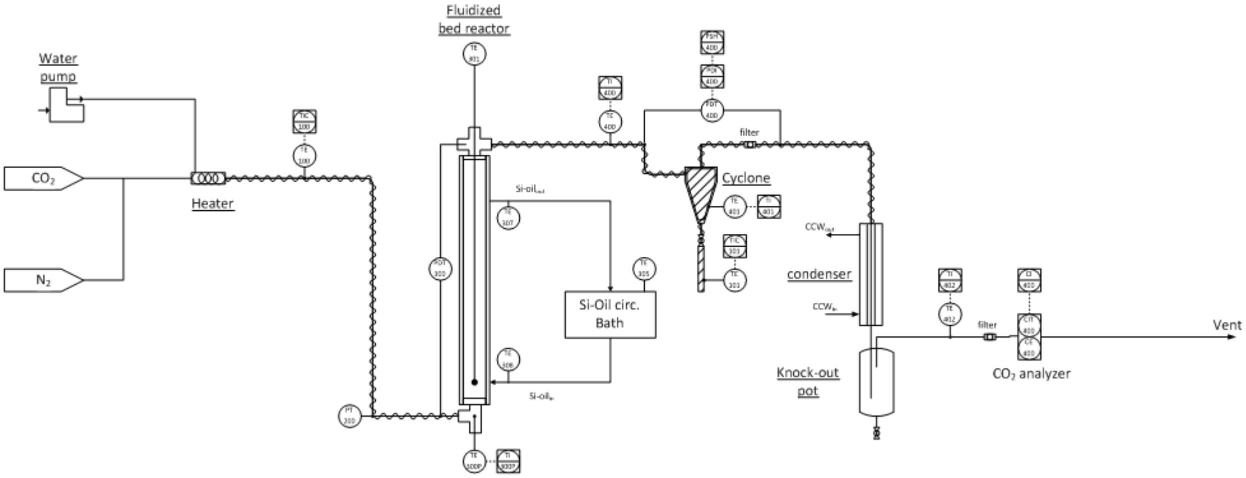

Scheme S2. Process flow diagram of the RTI's visual-fluidized bed reactor system (vFBR).

The system consisted of the gas feed manifold equipped with N_2 and CO_2 mass flow controllers and a water pump. The gas and water feed streams are merged at the heating coil where the water is heated and evaporated, producing a humid gas stream leaving the heater. The humid gas stream is introduced at the bottom of the jacketed 1" ID borosilicate glass (fluidized bed) reactor through a glass frit which serves as a gas distributor and support for sorbent. The reactor jacket is filled with silicone oil and temperature controlled by a circulation bath for both heating and cooling during the test. The reactor effluent leaves the reactor through the top and enters in a cyclone to remove any entrained solids. The gas stream is sent to a condenser to remove water and finally fed to a CO_2 analyzer to monitor the concentration during the experiment. The system is heat-traced to prevent water condensation and equipped with safety limits to mitigate unsafe conditions and equipment damage should an unexpected temperature or pressure excursion occurs. The reactor is loaded with 50 g of sorbent, which fills about 20-30% of the reactor volume in a typical run.

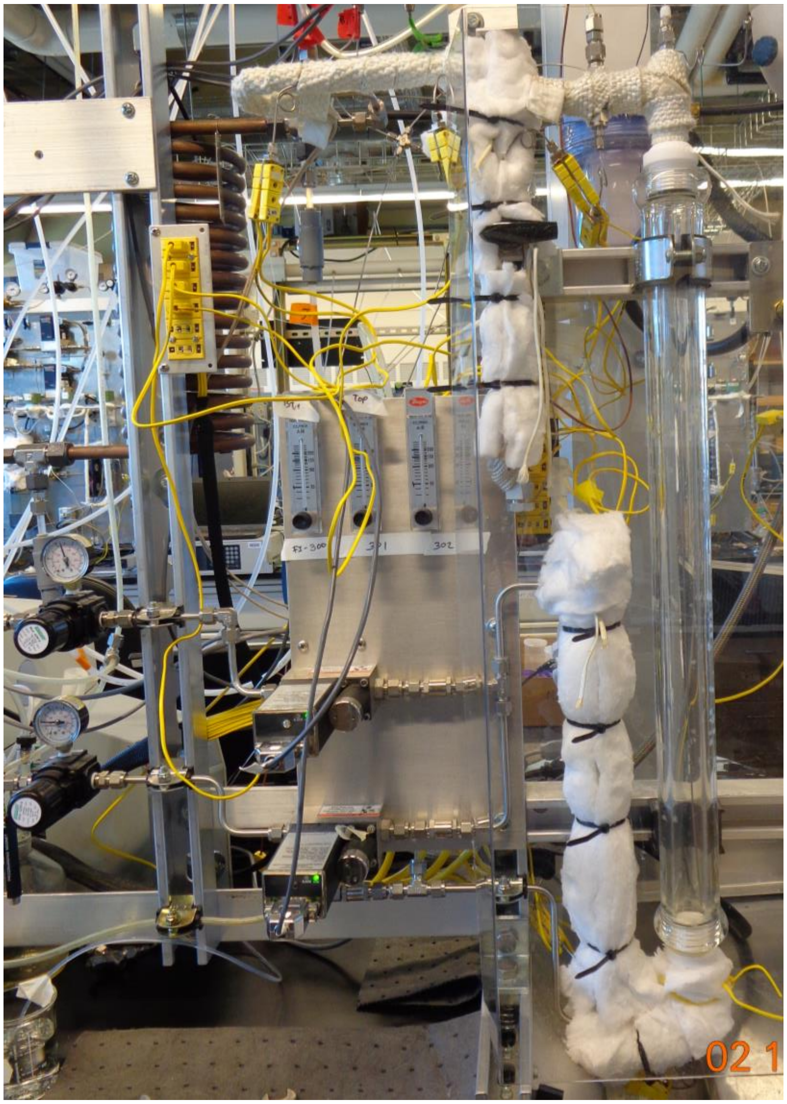

Figure S2. Image of the FBR.

2. Characterization

Table S1. MOF loading and surface area for selected MOF/SiO_2.

**MOF/SiO_2 material**	**MOF (wt%)**	**S_{BET} (m^2/g)**
(Cr)MIL-101(SO_3H)	40.0	865
(Cr)MIL-101(SO_3H)	19.1	486
(Cr)MIL-101(SO_3H)	4.9	258
(Zr)UiO-66(NH_2)	37.6	434
(Zr)UiO-66(NH_2)	4.2	243
(Zn)ZIF-8	35.1	346
(Zn)ZIF-8	4.6	241
(Cr)MIL-100	4.6	260
(Zr)PCN-222	7.9	297
(Zr)NU-1000	4.2	302

S_{BET} (SiO_2) = 256 m^2/g

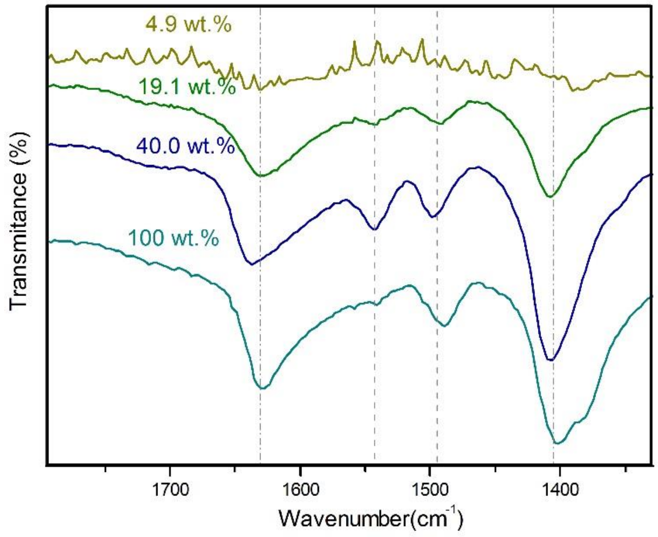

Figure S3. FTIR spectra for (Cr)MIL-101(SO_3H)/SiO_2 at varying loadings compared to pure MOF (100 wt.%)

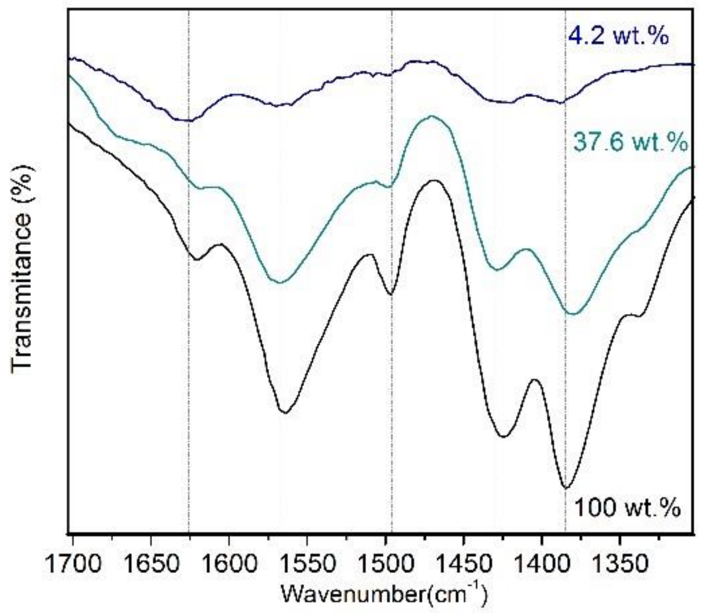

Figure S4. FTIR spectra for (Zr)UiO-66(NH_2)/SiO_2 at varying loadings compared to pure MOF (100 wt.%)

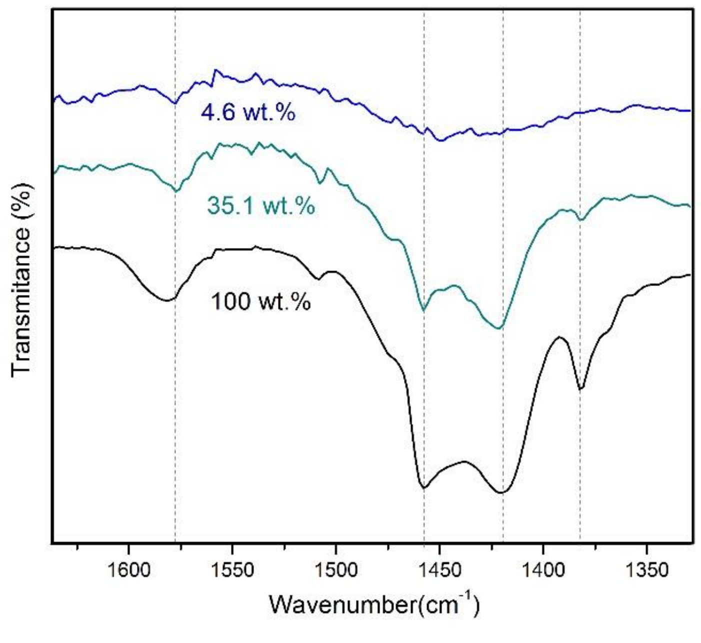

Figure S5. FTIR spectra for (Zn)ZIF-8/SiO_2 at varying loadings compared to pure MOF (100 wt.%)

Table S2. Resume of the performance and selected characteristics for polyamine coordinated to (Cr)MIL-101(SO_3H)/SiO_2 at varying loadings in a packed bed reactor under simulated flue gas conditions.

MOF (wt.%)	polyamine type	N (wt.%)[a]	S (wt.%)[a]	N : S molar	CO_2 (wt.%)[b]	Deactivation (%)[c]	CO_2:N molar[d]
**100**	TEPA	6.9	6.1	2.6	2.5	28	0.14
**40.0**	TEPA	5.1	1.2	9.6	3.4	18	0.26
**19.1**	TEPA	3.1	0.7	10.0	3.4	24	0.43
**19.1**	DETA	1.7	0.6	6.4	1.1	62	0.31

[a] Calculated by elementary analysis. [b] CO_2 adsorption capacity at the 1st cycle measured in a PBR under simulated flue gas conditions. [c] 10 cycles CO_2 adsorption deactivation. [d] Calculated considering one N of the polyamine coordinated to the Cr or sulfonic acid and then non-active for CO_2 capture.

Table S3. Resume of the performance and selected characteristics of two PEI/MOF/SiO_2 evaluated for CO_2 capture in a packed bed reactor under simulated flue gas conditions before and after 250-cycle run.

MOF	PEI (wt%)	MOF (wt%)	N (wt%) [fresh/used]	C (wt%) [fresh/used]	H (wt%) [fresh/used]	CO_2 Capacity (%)[a]	Deactivation (%)[b]
**ZIF-8**	-	4.6	1.66	3.79	0.82	-	-
	35	3.0	13.38/12.05	22.36/20.57	4.84/4.35	**12.5**	8
**ZIF-7**	-	4.9	1.63	4.46	0.68		
	35	3.2	13.09/11.87	22.24/20.26	4.60/4.38	**12.0**	12

[a]CO_2 capacity measured at the 50th cycle. [d] Deactivation calculated by difference between capacities at the 10th cycle and 250th cycle.

Table S4. Resume of the performance and selected characteristics for PEI/ZIF-8/SiO_2 evaluated for CO_2 capture in a packed bed reactor under simulated flue gas conditions and the presence of contaminants.

	N (wt%)	C (wt%)	H (wt%)	S (wt%)	CO_2 (%)[a]	Deactivation (%)[b]
**fresh**	13.38	22.36	4.84	**-**	**-**	-
**0 ppm**	12.05	20.57	4.35	-	12.5	1.7
**50 ppm SO_2**	11.74	19.69	4.7	1.8	12.41	31.1
**200 ppm SO**	9.1	16.0	4.33	6.3	12.86	85.7
**200 ppm NO_x**	12.5	20.6	4.5	-	13.29	12.8
**1% H_2S[d]**	11.3	18.3	5.5	0.4	10.6	1.45

[a] CO_2 adsorption capacity at the 10th cycle measured in a PBR under simulated flue gas conditions. [b] 100 cycles CO_2 adsorption deactivation. [c] Dry conditions were used because safety issues because H_2S cylinder also contains H_2.

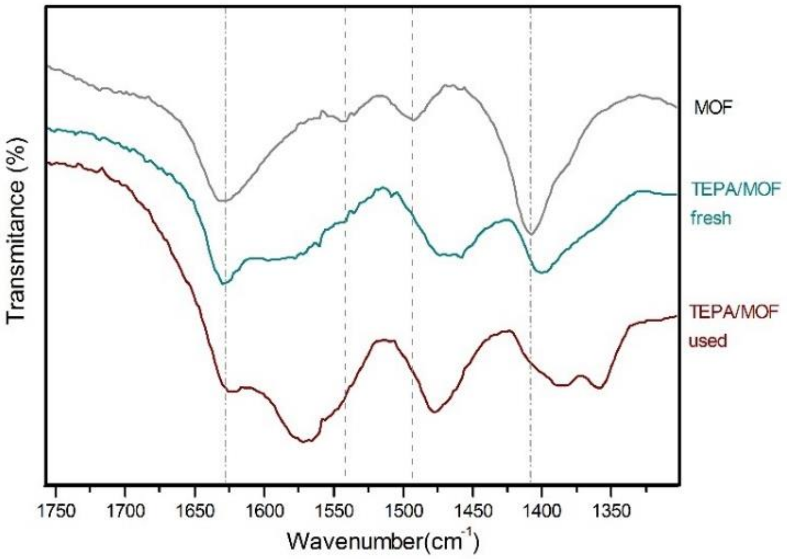

Figure S6. FTIR spectra of TEPA coordinated on bulk (Cr)MIL-101(SO_3H) before and after 10 cycle run in a packed bed reactor under simulated flue gas conditions.

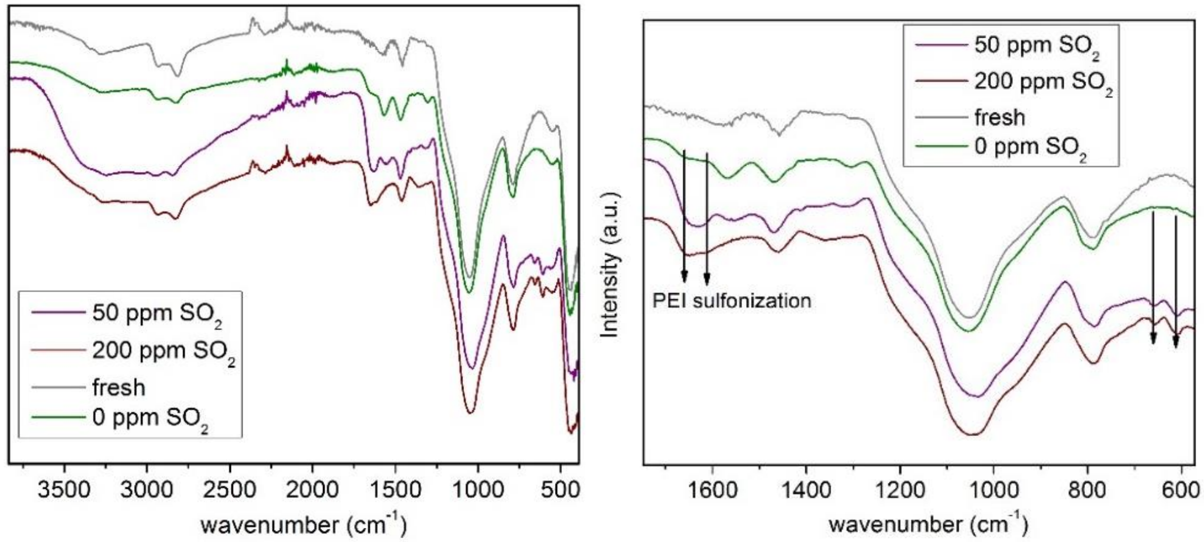

Figure S7. FTIR spectra for hybrid sorbent PEI/(Zn)ZIF-8/SiO_2 after 100 cycle test under the presence of 0, 50 and 200 ppm of SO_2.

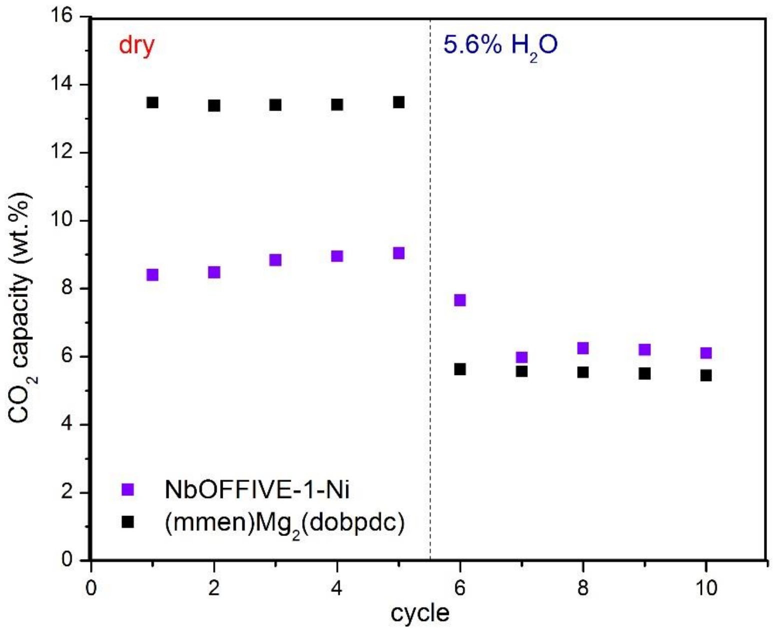

Figure S8. CO_2 capture for reported bulk MOFs under dry and wet conditions.

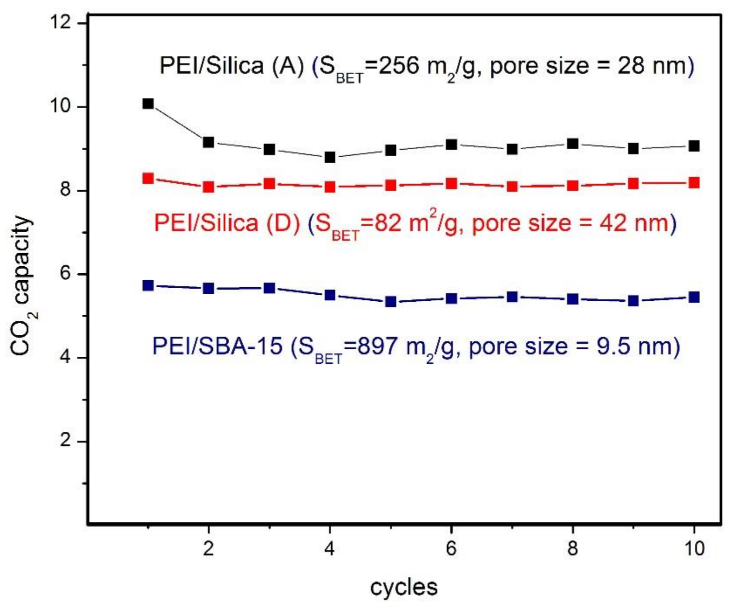

Figure S9. CO2 capture under simulated flue gas conditions for 35 wt.% PEI impregnated on Silica(A) (used in this work) compared to Silica(D) and SBA-15.

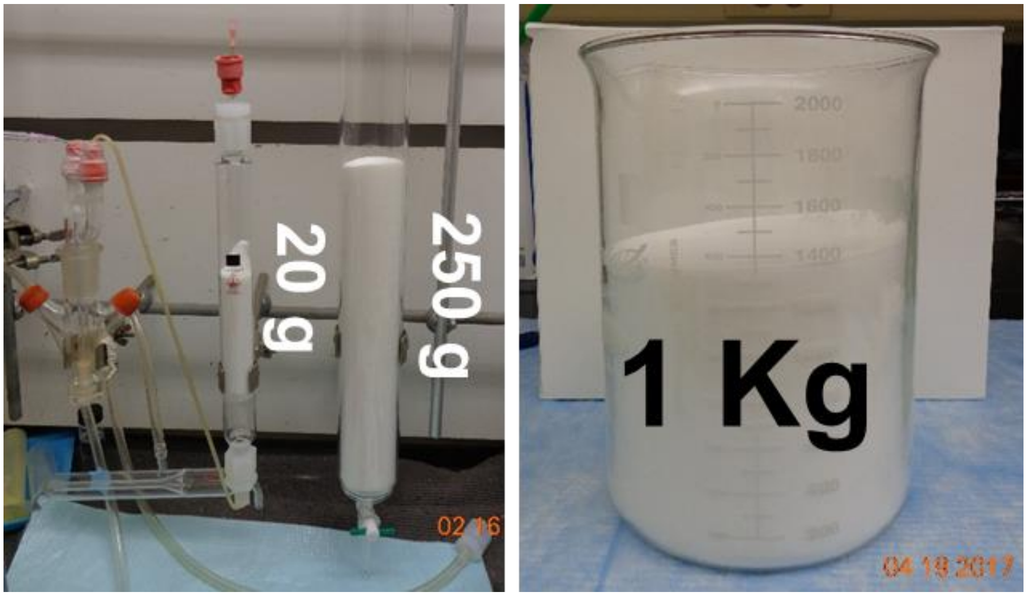

Figure S10. Scale up from gram to Kg scale of PEI/MOF/SiO_2 fluidized CO_2 sorbent.

	Compound	Commercial-Scale Cost ($/kg)	Cost per kg sorbent produced ($/kg)	Cost Ratio to Sorbent Production
Silica	Silicycle 150 Å	15.4	9.24	53.1%
			**+9.24**	**+53.1%**
MOF	Zinc Nitrate Hexahydrate	1.9	0.06	0.6%
	2-Methylimidazole	4.9	0.18	1.0%
	Triethylamine	1.5	0.11	0.7%
			**+0.35**	**+2.3%**
Solvents	Methanol	0.4	1.02	6.4%
	Chloroform	1.1	1.6	7.9%
			**+2.6**	**+14.3%**
Amine	600 MW PEI	22	7.7	30.3%
			**+7.7**	**+30.3%**
		**Total Cost**	**19.89**	**100%**

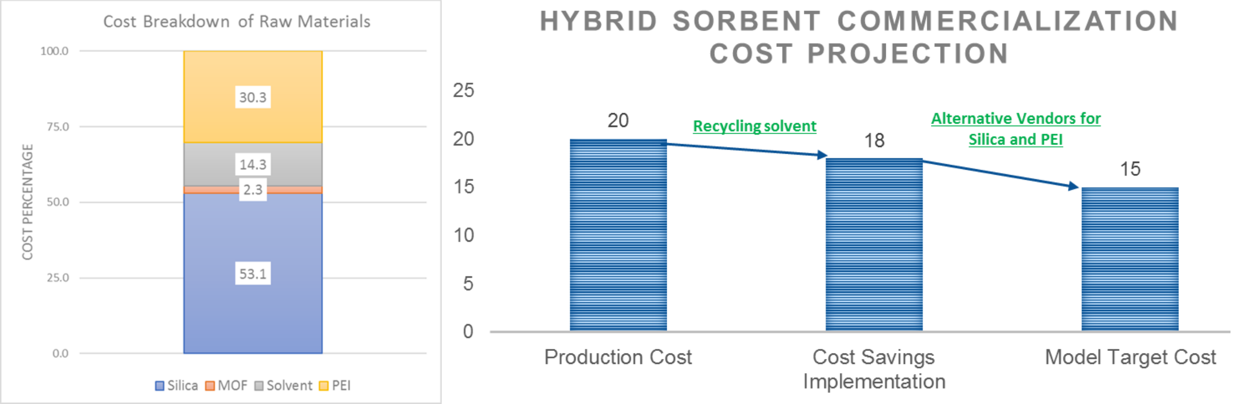

Figure S11. Resume of cost evaluation.

References

(1) Deria, P.; Bury, W.; Hupp, J. T.; Farha, O. K.: Versatile functionalization of the NU-1000 platform by solvent-assisted ligand incorporation. *Chemical Communications* **2014**, *50*, 1965-1968.

(2) McDonald, T. M.; Lee, W. R.; Mason, J. A.; Wiers, B. M.; Hong, C. S.; Long, J. R.: Capture of Carbon Dioxide from Air and Flue Gas in the Alkylamine-Appended Metal-Organic Framework mmen-Mg-2(dobpdc). *Journal of the American Chemical Society* **2012**, *134*, 7056-7065.

(3) Luz, I.; Soukri, M.; Lail, M.: Confining Metal–Organic Framework Nanocrystals within Mesoporous Materials: A General Approach via “Solid-State” Synthesis. *Chemistry of Materials* **2017**.

(4) Gómez-Gualdrón, D. A.; Moghadam, P. Z.; Hupp, J. T.; Farha, O. K.; Snurr, R. Q.: Application of Consistency Criteria To Calculate BET Areas of Micro- And Mesoporous Metal–Organic Frameworks. *Journal of the American Chemical Society* **2016**, *138*, 215-224.
